# Pulmonary tuberculosis epidemiology and genetics in Kazakhstan

**DOI:** 10.3389/fpubh.2024.1340673

**Published:** 2024-04-19

**Authors:** Dauren Yerezhepov, Aidana Gabdulkayum, Ainur Akhmetova, Ulan Kozhamkulov, Saule Rakhimova, Ulykbek Kairov, Gulnur Zhunussova, Ruslan Kalendar, Ainur Akilzhanova

**Affiliations:** ^1^Laboratory of Genomic and Personalized Medicine, Center for Life Sciences, National Laboratory Astana, Nazarbayev University, Astana, Kazakhstan; ^2^Laboratory of Bioinformatics and Systems Biology, Center for Life Sciences, National Laboratory Astana, Nazarbayev University, Astana, Kazakhstan; ^3^Institute of Genetics and Physiology, Almaty, Kazakhstan

**Keywords:** pulmonary tuberculosis, infectious disease, genetic predisposition, immune response, epidemiology

## Abstract

**Background:**

Tuberculosis (TB) is a major public health emergency in many countries, including Kazakhstan. Despite the decline in the incidence rate and having one of the highest treatment effectiveness in the world, the incidence rate of TB remains high in Kazakhstan. Social and environmental factors along with host genetics contribute to pulmonary tuberculosis (PTB) incidence. Due to the high incidence rate of TB in Kazakhstan, our research aimed to study the epidemiology and genetics of PTB in Kazakhstan.

**Materials and methods:**

1,555 participants were recruited to the case–control study. The epidemiology data was taken during an interview. Polymorphisms of selected genes were determined by real-time PCR using pre-designed TaqMan probes.

**Results:**

Epidemiological risk factors like diabetes (*χ*^2^ = 57.71, *p* < 0.001), unemployment (χ^2^ = 81.1, *p* < 0.001), and underweight-ranged BMI (<18.49, *χ*^2^ = 206.39, *p* < 0.001) were significantly associated with PTB. VDR FokI (rs2228570) and VDR BsmI (rs1544410) polymorphisms were associated with an increased risk of PTB. A/A genotype of the TLR8 gene (rs3764880) showed a significant association with an increased risk of PTB in Asians and Asian males. The G allele of the rs2278589 polymorphism of the MARCO gene increases PTB susceptibility in Asians and Asian females. VDR BsmI (rs1544410) polymorphism was significantly associated with PTB in Asian females. A significant association between VDR ApaI polymorphism and PTB susceptibility in the Caucasian population of Kazakhstan was found.

**Conclusion:**

This is the first study that evaluated the epidemiology and genetics of PTB in Kazakhstan on a relatively large cohort. Social and environmental risk factors play a crucial role in TB incidence in Kazakhstan. Underweight BMI (<18.49 kg/m2), diabetes, and unemployment showed a statistically significant association with PTB in our study group. FokI (rs2228570) and BsmI (rs1544410) polymorphisms of the VDR gene can be used as possible biomarkers of PTB in Asian males. rs2278589 polymorphism of the MARCO gene may act as a potential biomarker of PTB in Kazakhs. BsmI polymorphism of the VDR gene and rs2278589 polymorphism of the MARCO gene can be used as possible biomarkers of PTB risk in Asian females as well as VDR ApaI polymorphism in Caucasians.

## Introduction

Tuberculosis (TB) is an infectious disease caused by *Mycobacterium tuberculosis* (MTB) and is a major public health emergency in many countries, including Kazakhstan. Before the COVID-19 pandemic, TB was the world’s leading cause of death from a single infectious agent (1). The lowest incidence rate is registered in most high-income countries and is lower than 10 per 100,000 population, while the highest rate of 400 per 100,000 population per year is registered in eight countries. There are 30 countries burdened by TB and they build up to 80% of total cases worldwide with an average incidence rate of 183 cases per 100,000 population (2). Enormous research has been done to understand the epidemiology and pathophysiology of TB, and many programs put effort into the fight against TB, which has led to a decline in the number of cases in the new millennia. However, over 10 million new cases of active TB occur annually (3). The incidence rate and mortality of TB remain high since cases involving multi-drug (MDR) and extensive-drug resistant (XDR) strains of MTB are rising every year (1, 4).

Despite having the largest economy in the Central Asian region, Kazakhstan was transferred to the middle-income countries list at the beginning of the new century when Kazakhstan became one of the fastest-growing countries in the world (5). The healthcare system also rose, the increase in the quality of medical care along with the implementation of several anti-TB programs led to a tangible decrease in TB incidence rates and mortality, and an increase in treatment effectiveness. The incidence rate of pulmonary TB (PTB) in Kazakhstan has declined more than two times in the last two decades (from 171 in 2000 to 78 in 2022 per 100,000 population), and the TB mortality rate decreased from 7.4 to 1.5 deaths per 100,000 population. The effectiveness of treatment of TB patients in Kazakhstan reached one of the highest indicators in the world. In 2021, the treatment effectiveness of newly diagnosed patients with sensitive TB was 85.9%, and with drug resistance – 80.2% (6). The government putting an enormous effort into fighting against TB, but cases involving drug-resistant strains of MTB dramatically increased in Kazakhstan. At present time, multidrug-resistant TB (MDR-TB) constitutes almost 26% of primary TB and over 44% of retreatment cases in Kazakhstan (7). Currently, Kazakhstan is among the 30 high MDR-TB-burden countries in the world (1).

TB is a multifactorial disease and the development of which depends on many social and environmental factors. According to the World Health Organization (WHO), such risk factors include smoking, alcohol overconsumption, contact with TB patients, low body mass index (BMI, under 18.5 kg/m^2^), human immunodeficiency virus (HIV) co-infection, drug abuse, migration, and diabetes (8, 9). TB does not affect genders or age groups equally. Males are twice as susceptible to TB than women. However, pregnancy and maternal leave increase the susceptibility to TB in women (1, 8, 9). Social and environmental factors play a crucial role in TB incidence in low- and middle-income countries where socially vulnerable individuals constitute a major part of TB cases (10).

By estimation, *M. tuberculosis* has co-evolved with human populations for between 40,000 and 70,000 years (11) resulting in the evolutionary adaptation of the human organism to eliminate the pathogen at the very first encounter or hold the invaded bacteria from multiplication inside the body by the cooperation of innate and adaptive immunity for several months or even for a lifetime. This state is called latent TB (LTB). Individuals with latent TB are infected with MTB, do not show any clinical symptoms, and do not have the disease, but are at higher risk of transitioning to an active form of TB in the future (4). Globally from 25 to 30% of the general population has latent TB, and only up to 10% of them will have active forms of TB (12). This data implies that epidemiological risk factors and host genetics take part in the shift from LTB to an active form of the disease (11, 13).

The immune response to MTB is induced when macrophages recognize certain conserved structures in the cell wall of the invading pathogen with their pattern recognition receptors (PRRs). Toll-like receptors (TLRs) are an important class of PRRs that recognize pathogen-associated molecular patterns (PAMPs). The recognition process activates the expression of many immune response genes encoding various types of pro- and anti-inflammatory molecules, including cytokines (interleukin 1β – IL-1β, and interferon-γ – IFN-γ, etc.), transmembrane receptors (macrophage receptor with collagenous structure – MARCO, and class A scavenger receptors – SRA), monooxygenases (CYP27B1), vitamin D receptor (VDR), and intracellular mediators (nitric oxide – NO) (14–16).

The role of genetics in susceptibility to TB was the subject of extensive research. Initial research was done by twin studies in the mid-40s of the twentieth century by Kallmann and Reisner (17), then Comstock performed a re-analysis 45 years later (18). Later, new methods of genetic engineering allowed researchers to test the genetic susceptibility to TB by animal models (19–21). Since an enormous number of genes are involved in the immune response, numerous point mutations or single nucleotide polymorphisms (SNPs) in many candidate genes were studied, and with the development of microarray technologies, many populations were tested by genome-wide association studies (GWAS) (22–27). However, evolutionary differences in the distribution of genotypes and their frequencies in the different populations give rise to a lack of consistency and low replicability of these research works (28).

The initial step of the immune response is very important. TLRs are important in inducing multidirectional activation of inflammatory responses during MTB infection and play a key role in the development of antigen-specific adaptive immunity (29). Proper recognition of PAMPs by cell-surface expressing TLRs, several of which are required to build heterodimers, is very important for the activation of all players involved in the immune response against MTB. Any alterations in nucleotide sequences of genes encoding TLRs could lead to structural changes and, subsequently, decrease the functionality of these receptors, and affect the whole immune response chain (30). Many research works tested the association of various genetic polymorphisms of TLR genes with TB in many populations, and these works also showed some level of inconsistency (23, 30, 31).

The role of pro- and anti-inflammatory molecules like cytokines, transmembrane receptors, vitamin D, and intracellular mediators in the immune response against MTB is very important (14). Macrophages and neutrophils encountered with MTB produce interleukins that can act as innate immune modulators and are very important during the early stages of infection (32). Interferons are released during MTB infection as a part of the innate immune response and play their part in controlling the infection (33). Transmembrane receptors like SRA and MARCO bind with MTB by cysteine-rich domains and initiate phagocytosis (34). Nitric oxide is an intracellular mediator that directly inhibits the growth of bacteria (35).

The importance of vitamin D in the modulation of the immune response against various pathogens is proven by many research works (36, 37). Recognition of PAMPs by TLRs induces expression of the vitamin D receptor (VDR) gene which is required for binding and transferring the 1,25-dihydroxyvitamin D (25-(OH)D), the active form of vitamin D, into the nucleus and performing its function (38). Any gene variations of VDR might affect the binding of 1,25-dihydroxyvitamin D with its receptor and alter the immune response.

Since TB is a multifactorial infectious disease and the incidence rate in Kazakhstan remains high, the aim of our research was to study the epidemiology and genetics of pulmonary tuberculosis in Kazakhstan.

## Materials and methods

### Study subjects

A case–control study included individuals diagnosed with active primary pulmonary TB. The group of controls consisted of participants with no family history (first-degree relatives) of pulmonary tuberculosis. Diagnosis of the case group was confirmed by radiographic evidence of TB cavitation and positive laboratory data. The case group participants were recruited at local TB dispensaries. The control subjects were matched by gender, age, and ethnicity, and had clear chest X-rays not older than 3 months before the recruitment date.

All subjects were older than 18 years, with available clinical data. The participants’ clinical information was obtained from the Unified National Electronic Healthcare System (UNEHS).

UNEHS databases provide several data elements and variables including ID and its registration in any sub-databases, information on medical organizations that registered ID, demographic data (birth date, gender, ethnicity, education, residency type), outcomes, all diagnoses with classification, stay in the medical organization, and prescription of medication. The National Registry of Tuberculosis Patients (NRTP) registers all patients under ambulatory follow-up in a designated polyclinic. All contacts of TB patients (household, colleagues, etc.) are under mandatory 6-month follow-up and this information is registered in NRTP. The medical data of the UNEHS database that we used to define the control subjects was (1) TB status (patient or contact person), (2) BCG vaccination status, (3) diabetes, (4) cancers, (5) chronic upper respiratory tract diseases, (6) other immune-compromising diseases, (7) medical prescriptions that affect body mass index (BMI). Additional questions for several medical conditions (diabetes, cancers, cardiovascular diseases, varicose veins, allergies, etc.) and epidemiological risk factors were included in the questionnaire (Supplementary material).

Eligible participants filled out structured sociodemographic and clinical questionnaires during an in-person interview. Anthropometric indicators, including height and weight, were measured during an interview.

We could not identify the latent TB status of the participants since all had been vaccinated with BCG which we indicated in the limitations of the current research. Additionally, the Interferon-Gamma Release Assay (IGRA) blood test costs over 50 USD per person and we could not perform it due to financial limitations.

The study was conducted in accordance with the Declaration of Helsinki and approved by the Local Ethics Committee of the Private Institution “National Laboratory Astana” (01–2020, June 26, 2020). All subjects signed a written informed consent form (Supplementary material).

### DNA isolation

Blood was collected into tubes containing K_2_EDTA. DNA was extracted using the QIAamp DNA Mini extraction Kit (Qiagen GmbH, Germany) and Illustra blood genomicPrep Mini Spin Kit (Cytiva, Danaher, DC, United States), according to the manufacturers’ protocols. The quality and quantity of the DNA were evaluated using the NanoDrop-2000 UV (Thermo Fisher Scientific, Waltham, MA, United States) spectrophotometer. The exact quantity before dilution was assessed using Qubit BR Assay Kit (Thermo Fisher Scientific, Waltham, MA, United States) on a Qubit v2.0 fluorometer (Thermo Fisher Scientific, Waltham, MA, United States). The integrity of DNA was tested on 1% agarose gel electrophoresis running for 30 min at 120 V in TAE buffer. Gel was visualized on a GelDoc imaging system (Bio-Rad, United States). DNA was refrigerated at −20°C until further use.

### Genotyping

The recognition of MTB by macrophages induces the activation of several genes involved in the initial stages of the immune response. Genetic variations in these genes can have an impact on the induction of particular members of the immune response chain and can cause alterations in immune response. From the literature search, we chose the most significant polymorphisms (12, 13, 15, 21–23, 27, 30, 33, 34), which showed statistical significance in many populations, and thus, had a lower level of inconsistency. Polymorphisms of the following genes were used for genotyping: VDR (rs2228570, rs731236, rs1544410, rs7975232), IL1B (rs16944), IFG (rs2430561), MARCO (rs2278589), NOS2 (rs2779248), TLR2 (rs1898830), and TLR8 (rs3764880). Detailed information on SNPs is presented in Supplementary material.

The gene polymorphisms were determined by allelic discrimination real-time polymerase chain reaction (RT-PCR) using pre-designed TaqMan™ probes (Thermo Fisher Scientific, Foster City, CA, United States) on 7900 HT Fast Real-Time PCR System following the manufacturer’s protocol. Ten microliter reaction mixture consisted of 5 uL of 2× TaqMan™ Genotyping master mix (Thermo Fisher Scientific, Foster City, CA, United States), 0.25 uL of 40× TaqMan™ Probe (Thermo Fisher Scientific, Foster City, CA, United States) or 0.5 uL of 20× TaqMan™ Probe (Thermo Fisher Scientific, Foster City, CA, United States). A total of 10 ng of genomic DNA was added to the reaction. Two microliter of nuclease-free water was added to negative control which was included in each set of reactions. RT-PCR program consisted of 10 min incubation at 50°C, denaturation at 95°C for 3 min, followed by 40 cycles of 92°C for 15 s and 60°C for 1 min with automatic scanning after each cycle.

The distribution of the genotypes was analyzed using SDS software (v2.4, Thermo Fisher Scientific, Foster City, CA, United States). The software distributes each of the genotypes according to the obtained signals of a preloaded set of detectors. To avoid biases in the genotype distribution, the genotyping of each sample was repeated three times.

### Statistical analysis

Quantitative variables were expressed as a result (± standard deviation) with normal distribution. The Hardy–Weinberg equilibrium test was applied separately for all subjects, case, and control groups. 2×2 association between the risk of pulmonary TB and epidemiological risk factors was analyzed using the Pearson χ^2^ test. The association between the risk of pulmonary TB and selected polymorphisms was evaluated using multimodal (genotypic, dominant, recessive, and overdominant) logistic regression and assessed with the ORs and their corresponding 95% CIs. We defined the models as follows: genotypic (AA vs. Aa vs. aa), dominant (AA vs. Aa + aa), recessive (AA + Aa vs. aa), and overdominant (AA + aa vs. Aa), where the major and the minor alleles are A and a, respectively. All the tests were 2-sided, with a significance level of *p* < 0.05, and were estimated using SPSS 25 (IBM, Armonk, NY, United States) software.

## Results

### Study group

**Figure 1:**
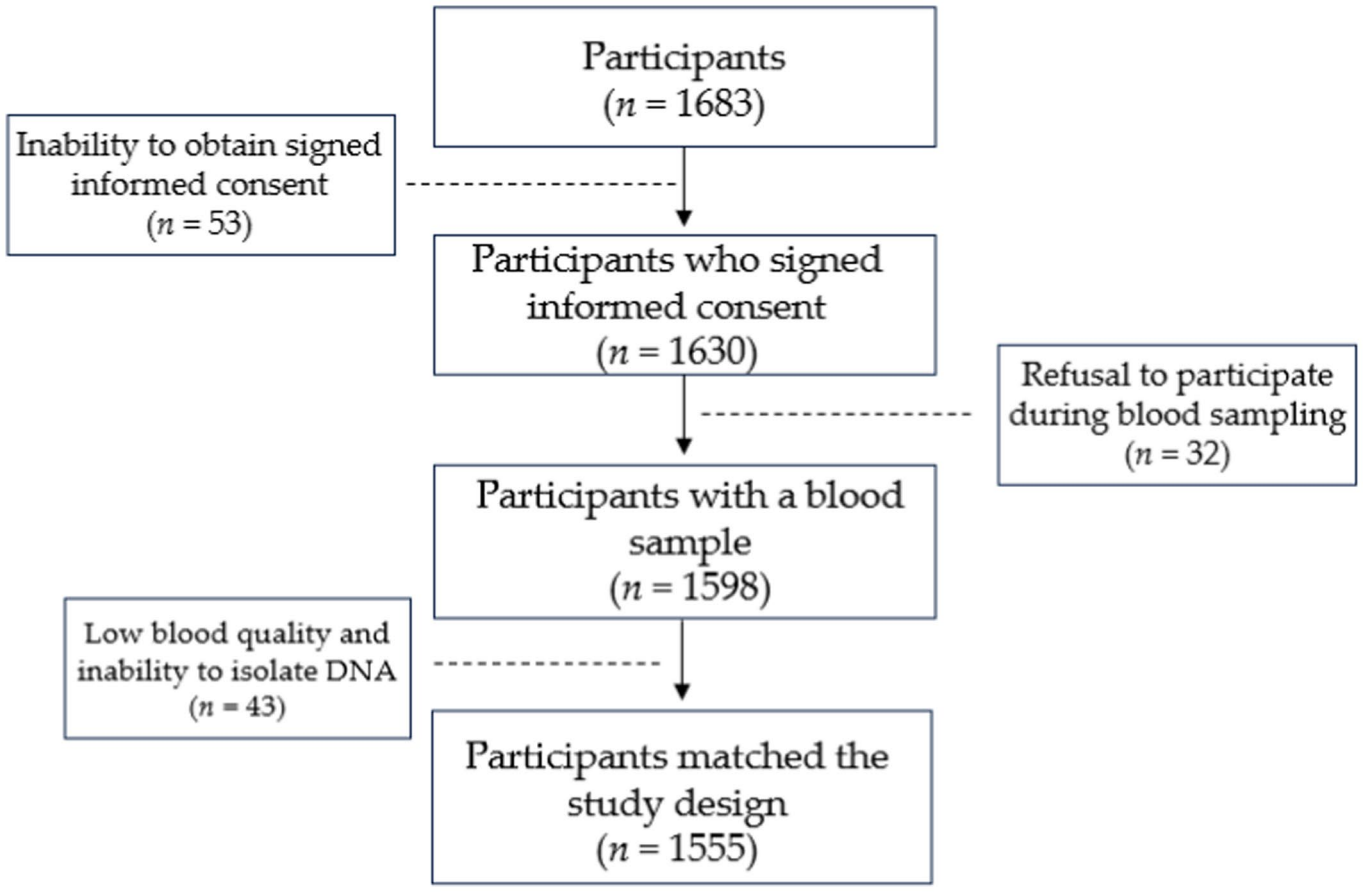
Illustration of study design.

Sixteen hundred and eighty-three individuals were recruited for the study. Fifty-three individuals were excluded due to the inability to obtain signed informed consent. During the blood sampling, 32 individuals refused to provide blood samples. Another 43 individuals from both groups were removed due to low blood quality and inability to perform DNA isolation. Five hundred and fifty-seven individuals diagnosed with active primary PTB and 998 participants with no family history of TB constituted the final group of 1,555 participants. The responder rate in the case and control groups was 59.4 and 96.3%, respectively. Study design is shown in Figure 1.

### Epidemiology of pulmonary TB in Kazakhstan

#### Demographic characteristics and epidemiological data

At enrollment, the median age was 39.0 ± 13.84 years (18–76), median height was 167.07 ± 8.46 cm, weight 65.41 ± 13.28 kg and BMI 23.40 ± 4.40. Slightly over half of the participants were females (53.3%). Almost four-fifths were Asians (79.8%). The study group consisted of 557 cases of primary pulmonary TB and 998 controls with no family history of TB (Table 1). All participants had BCG scars.

**Table 1:** Demographic and epidemiological data of study participants.

Details	All (*n* = 1,555)	Case (*n* = 557)	Controls (*n* = 998)
Age, mean ± *SD*, years	39.0 ± 13.84	35.88 ± 13.05	40.91 ± 13.46
Height, mean ± *SD*, cm	167.07 ± 8.46	167.22 ± 8.85	166.99 ± 8.24
Weight, mean ± *SD*, kg	65.41 ± 13.28	59.60 ± 11.26	68.66 ± 13.22
BMI, mean ± *SD*, kg/m2	23.40 ± 4.40	21.29 ± 3.64	24.61 ± 4.45
Gender, *n* (%)			
Male	726 (46.7)	308 (55.2)	418 (41.9)
Female	829 (53.3)	249 (44.7)	580 (58.1)
Ethnicity, *n* (%)			
Asian	1242 (79.8)	447 (80.3)	795 (79.7)
Caucasian	313 (20.2)	110 (19.7)	203 (20.3)
Risk factors, *n* (%)			
Residence			
Urban	687 (44.2)	248 (50.0)	439 (44.0)
Rural	868 (55.8)	248 (50.0)	559 (56.0)
Employment			
No	835 (53.7)	384 (68.9)	451 (45.2)
Yes	720 (46.3)	173 (31.1)	547 (54.8)
Alcohol consumption			
No	1528 (98.3)	544 (97.7)	984 (98.6)
Yes	27 (1.7)	13 (2.3)	14 (1.4)
BMI, kg/m^2^			
<18.49	162 (10.4)	124 (22.3)	38 (3.80)
18.50 > <24.99	917 (59.0)	359 (64.5)	558 (55.9)
>25.00	476 (30.6)	74 (13.3)	402 (40.3)
Diabetes			
No	1500 (96.5)	511 (91.7)	989 (99.1)
Yes	55 (3.5)	46 (8.3)	9 (0.9)
Contact with TB			
No	1523 (97.9)	525 (94.3)	998 (100)
Yes	32 (2.1)	32 (5.7)	0 (0)
Smoking			
No	1331 (85.6)	466 (83.7)	865 (86.7)
Yes	224 (14.4)	91 (16.3)	133 (13.3)
Drug abuse			
No	1552 (99.8)	554 (99.5)	998 (100)
Yes	3 (0.2)	3 (0.5)	0 (0)
HIV			
No	1546 (99.4)	549 (98.6)	997 (99.9)
Yes	9 (0.6)	8 (1.4)	1 (0.1)
Maternity Leave (*n* = 829), *n* (%)			
No	819 (98.8)	239 (96)	580 (100)
Yes	10 (1.2)	10 (4)	0 (0)

BMI, body mass index; HIV, human immunodeficiency virus; SD, standard deviation; TB, tuberculosis.

### Epidemiological risk factors of pulmonary TB

Epidemiological risk factors of pulmonary TB are presented in Table 1. Over 55% of the case group consisted of males, and little less than three-fifths of the control group were females (58.1%). 55.8% of the study group were living in rural locations, 835 (53.7%) participants were unemployed, 1.7% of the participants consumed alcohol regularly, 224 participants were active smokers, 55 participants (3.5%) had diabetes with prevalence in the case group, 32 participants were in contact with TB patients, and all of them are in the case group, as 3 drug abusers. Nine participants had human immunodeficiency virus (HIV) and 8 of them were in the case group. 59% of participants were in normal BMI ranges (18.50 > <24.99), over 30% were overweight or obese, and a little over 10% were in underweight BMI ranges with prevalence in the case group (124 vs. 38 in controls). Out of 829 recruited females, 10 were on maternity leave (1.2%), and all were diagnosed with primary PTB.

Ethnicity, residence, alcohol consumption, and smoking showed no significant association with PTB in the studied population (Table 2). There were no participants who had contact with TB patients, no drug abusers in the overall control group, and no participants on maternity leave among females in the control group. In addition, a single participant in the control group had HIV (vs. eight in the cases). Due to the lack or low number of participants with corresponding risk factors, the contribution of contact with TB patients, drug abuse, HIV, and maternity leave, cannot be estimated. There were dramatic differences in case and control groups regarding diabetes, employment, and underweight-ranged BMI. Diabetes (χ^2^ = 57.71, *p* < 0.001), unemployment (χ^2^ = 81.1, *p* < 0.001), and underweight BMI (<18.49, χ^2^ = 206.39, *p* < 0.001) are strongly associated with the PTB.

**Table 2:** Association between PTB and socioeconomic and environmental risk factors (*n* = 1,555).

Risk factors		Case, *n* (%)	Control, *n* (%)	*χ* ^2^	*p*-value
Ethnicity	Asian	447 (80.3)	795 (79.7)	0.08	0.78
	Caucasian	110 (19.7)	203 (20.3)		
Diabetes	No	511 (91.7)	989 (99.1)	57.71	<0.001
	Yes	46 (8.3)	9 (0.9)		
Drug abuse	No	554 (99.5)	998 (100)	5.39	0.02
	Yes	3 (0.5)	0 (0)		
Contact with TB	No	525 (94.3)	998 (100)	58.54	<0.001
	Yes	32 (5.7)	0 (0)		
Residence	Urban	248 (45.5)	439 (44)	0.042	0.84
	Rural	309 (55.5)	559 (56)		
Alcohol consumption	No	544 (97.7)	984 (98.6)	1.82	0.18
	Yes	13 (2.3)	14 (1.4)		
Smoking	No	466 (83.7)	865 (86.7)	2.63	0.11
	Yes	91 (16.3)	133 (13.3)		
HIV	No	543 (98.6)	997 (99.9)	11.09	0.001
	Yes	8 (1.4)	1 (0.1)		
Employment	No	384 (68.9)	451 (45.2)	81.1	<0.001
	Yes	173 (31.1)	547 (54.8)		
BMI, kg/m^2^	<18.49	124 (22.3)	38 (3.8)	206.39	<0.001
	18.5 > <24.99	359 (64.5)	558 (55.9)		
	>25.00	74 (13.3)	402 (40.3)		
Maternity Leave (females, *n* = 829)	No	239 (96)	580 (100)	23.58	<0.001
	Yes	10 (4)	0 (0)		

BMI, body mass index; HIV, human immunodeficiency virus; TB, tuberculosis; *χ*^2^, Pearson’s chi square.

### Genetics of pulmonary tuberculosis in Kazakhstan

#### Genes and polymorphisms

All samples were successfully genotyped for SNPs. All SNPs were verified by triple repeat, and no deviations were detected. Only 4 individuals (0.26%) had the A/A genotype (rs1898830) of the TLR2 gene. Rest polymorphisms had more than 5% of minor allele frequency. According to the Hardy–Weinberg Equilibrium (HWE) test, genotype frequencies of TaqI, ApaI, BsmI, IL1B, MARCO, and NOS2 did not deviate from the expected frequency values. However, FokI, IFG, TLR2, and TLR8 polymorphisms deviated from HWE.

### Association between pulmonary tuberculosis and polymorphisms in the studied genes

The association between pulmonary tuberculosis and studied polymorphisms was evaluated through various models: genotypic, dominant, recessive, and overdominant, with no adjustment (crude analysis) and adjustment by gender (where applicable), age, BMI, smoking, alcohol consumption, and diabetes. Since the control group lacked or had a small number of participants with risk factors like contact with TB patients, drug abuse, HIV, and maternity leave, these risk factors were excluded from the adjustment model. The association between pulmonary tuberculosis and studied polymorphisms is shown in Table 3.

**Table 3:** Association between PTB and studied polymorphisms (*n* = 1,555).

Polymorphisms	Model	Genotype	Case *n* (%)	Control, *n* (%)	C-OR (95% CI)	*p*-value	A-OR (95% CI)^*^	*p*-value
VDR FokI	Gen	G/G	240 (43.1)	405 (40.6)	1.00	0.18	1.00	0.1
		A/G	233 (41.8)	464 (46.5)	1.18 (0.94–1.48)		1.25 (0.97–1.61)	
		A/A	84 (15.1)	129 (12.9)	0.91 (0.66–1.25)		0.90 (0.63–1.28)	
	Dom	G/G	240 (43.1)	405 (40.6)	1.00	0.34	1.00	0.23
		A/G-A/A	317 (56.9)	593 (59.4)	1.11 (0.90–1.37)		1.15 (0.91–1.46)	
	Rec	G/G-A/G	473 (84.9)	869 (87.1)	1.00	0.24	1.00	0.19
		A/A	84 (15.1)	129 (12.9)	0.84 (0.62–1.12)		0.80 (0.57–1.11)	
	Overdom	G/G-A/A	324 (58.2)	534 (53.5)	1.00	0.08	1.00	**0.04**
		A/G	233 (41.8)	464 (46.5)	1.21 (0.98–1.49)		**1.29 (1.02–1.63)**	
VDR TaqI	Gen	A/A	313 (56.2)	565 (56.6)	1.00	0.61	1.00	0.47
		A/G	207 (37.2)	379 (38)	1.01 (0.82–1.26)		1.00 (0.78–1.28)	
		G/G	37 (6.6)	54 (5.4)	0.81 (0.52–1.26)		0.74 (0.45–1.21)	
	Dom	A/A	313 (56.2)	565 (56.6)	1.00	0.87	1.00	0.74
		A/G-G/G	244 (43.8)	433 (43.4)	0.98 (0.80–1.21)		0.96 (0.76–1.22)	
	Rec	A/A-A/G	520 (93.4)	944 (94.6)	1.00	0.33	1.00	0.22
		G/G	37 (6.6)	54 (5.4)	0.80 (0.52–1.24)		0.74 (0.45–1.20)	
	Overdom	A/A-G/G	350 (62.8)	619 (62)	1.00	0.75	1.00	0.8
		A/G	207 (37.2)	379 (38)	1.04 (0.84–1.28)		1.03 (0.81–1.31)	
VDR ApaI	Gen	C/C	161 (28.9)	312 (31.3)	1.00	0.56	1.00	0.49
		A/C	284 (51)	483 (48.4)	0.88 (0.69–1.12)		0.85 (0.65–1.11)	
		A/A	112 (20.1)	203 (20.3)	0.94 (0.69–1.26)		0.92 (0.66–1.28)	
	Dom	C/C	161 (28.9)	312 (31.3)	1.00	0.33	1.00	0.27
		A/C-A/A	396 (71.1)	686 (68.7)	0.89 (0.71–1.12)		0.87 (0.67–1.12)	
	Rec	C/C-A/C	445 (79.9)	795 (79.7)	1.00	0.91	1.00	0.92
		A/A	112 (20.1)	203 (20.3)	1.01 (0.78–1.31)		1.01 (0.76–1.36)	
	Overdom	C/C-A/A	273 (49)	515 (51.6)	1.00	0.33	1.00	0.28
		A/C	284 (51)	483 (48.4)	0.90 (0.73–1.11)		0.88 (0.70–1.11)	
VDR BsmI	Gen	C/C	324 (58.2)	543 (54.4)	1.00	0.11	1.00	0.06
		C/T	207 (37.2)	385 (38.6)	1.11 (0.89–1.38)		1.20 (0.94–1.54)	
		T/T	26 (4.7)	70 (7)	1.61 (1.00–2.57)		1.74 (1.04–2.92)	
	Dom	C/C	324 (58.2)	543 (54.4)	1.00	0.15	1.00	**0.049**
		C/T–T/T	233 (41.8)	455 (45.6)	1.17 (0.95–1.44)		**1.27 (1.00–1.61)**	
	Rec	C/C-C/T	531 (95.3)	928 (93)	1.00	0.06	1.00	0.6
		T/T	26 (4.7)	70 (7)	1.54 (0.97–2.45)		1.62 (0.97–2.69)	
	Overdom	C/C-T/T	350 (62.8)	613 (61.4)	1.00	0.58	1.00	**0.02**
		C/T	207 (37.2)	385 (38.6)	1.06 (0.86–1.32)		**1.14 (0.90–1.45)**	
IL1B	Gen	G/G	180 (32.3)	360 (36.1)	1.00	0.22	1.00	0.66
		A/G	278 (49.9)	486 (48.7)	0.87 (0.69–1.10)		0.96 (0.74–1.25)	
		A/A	99 (17.8)	152 (15.2)	0.77 (0.56–1.05)		0.85 (0.60–1.21)	
	Dom	G/G	180 (32.3)	360 (36.1)	1.00	0.13	1.00	0.58
		A/G-A/A	377 (67.7)	638 (63.9)	0.85 (0.68–1.05)		0.93 (0.73–1.19)	
	Rec	G/G-A/G	458 (82.2)	846 (84.8)	1.00	0.19	1.00	0.39
		A/A	99 (17.8)	152 (15.2)	0.83 (0.63–1.10)		0.87 (0.64–1.19)	
	Overdom	G/G-A/A	279 (50.1)	512 (51.3)	1.00	0.65	1.00	0.91
		A/G	278 (49.9)	486 (48.7)	0.95 (0.77–1.17)		1.01 (0.80–1.28)	
IFN	Gen	T/T	312 (56)	525 (52.6)	1.00	0.25	1.00	0.8
		A/T	205 (36.8)	381 (38.2)	1.10 (0.89–1.38)		1.05 (0.82–1.34)	
		A/A	40 (7.2)	92 (9.2)	1.37 (0.92–2.03)		1.15 (0.74–1.79)	
	Dom	T/T	312 (56)	525 (52.6)	1.00	0.2	1.00	0.6
		A/T-A/A	245 (44)	473 (47.4)	1.15 (0.93–1.41)		1.07 (0.84–1.35)	
	Rec	T/T-A/T	517 (92.8)	906 (90.8)	1.00	0.16	1.00	0.58
		A/A	40 (7.2)	92 (9.2)	1.31 (0.89–1.93)		1.13 (0.73–1.74)	
	Overdom	T/T-A/A	352 (63.2)	617 (61.8)	1.00	0.59	1.00	0.82
		A/T	205 (36.8)	381 (38.2)	1.06 (0.86–1.31)		1.03 (0.81–1.31)	
MARCO	Gen	A/A	463 (83.1)	773 (77.5)	1.00	**0.03**	1.00	**0.01**
		A/G	88 (15.8)	212 (21.2)	**1.44 (1.10–1.90)**		**1.57 (1.15–2.14)**	
		G/G	6 (1.1)	13 (1.3)	**1.30 (0.49–3.44)**		**1.73 (0.59–5.10)**	
	Dom	A/A	463 (83.1)	773 (77.5)	1.00	**<0.01**	1.00	**< 0.01**
		A/G-G/G	94 (16.9)	225 (22.6)	**1.43 (1.10–1.87)**		**1.58 (1.17–2.13)**	
	Rec	A/A-A/G	551 (98.9)	985 (98.7)	1.00	0.7	1.00	0.39
		G/G	6 (1.1)	13 (1.3)	1.21 (0.46–3.21)		1.59 (0.54–4.67)	
	Overdom	A/A-G/G	469 (84.2)	786 (78.8)	1.00	**<0.01**	1.00	**< 0.01**
		A/G	88 (15.8)	212 (21.2)	**1.44 (1.09–1.89)**		**1.55 (1.14–2.12)**	
NOS2	Gen	C/C	266 (47.8)	537 (53.8)	1.00	0.056	1.00	0.08
		C/T	233 (41.8)	379 (38)	0.81 (0.65–1.00)		1.57 (1.15–2.14)	
		T/T	58 (10.4)	82 (8.2)	0.70 (0.49–1.01)		1.73 (0.59–5.10)	
	Dom	C/C	266 (47.8)	537 (53.8)	1.00	**0.02**	1.00	0.08
		C/T–T/T	291 (52.2)	461 (46.2)	**0.78 (0.64–0.97)**		1.58 (1.17–2.13)	
	Rec	C/C-C/T	499 (89.6)	916 (91.8)	1.00	0.15	1.00	0.06
		T/T	58 (10.4)	82 (8.2)	0.77 (0.54–1.10)		1.59 (0.54–4.67)	
	Overdom	G/G-T/T	324 (58.2)	619 (62)	1.00	0.14	1.00	0.49
		G/T	233 (41.8)	379 (38)	0.85 (0.69–1.05)		1.55 (1.14–2.12)	
TLR2	Gen	G/G	517 (92.8)	955 (95.7)	1.00	**0.03**	1.00	**0.03**
		A/G	37 (6.6)	42 (4.2)	**0.61 (0.39–0.97)**		**0.55 (0.33–0.91)**	
		A/A	3 (0.5)	1 (0.1)	**0.18 (0.02–1.74)**		**0.21 (0.02–2.51)**	
	Dom	G/G	517 (92.8)	955 (95.7)	1.00	**0.02**	1.00	**0.01**
		A/G-A/A	40 (7.2)	43 (4.3)	**0.58 (0.37–0.91)**		**0.53 (0.32–0.87)**	
	Rec	G/G-A/G	554 (99.5)	997 (99.9)	1.00	0.11	1.00	0.21
		A/A	3 (0.5)	1 (0.1)	0.19 (0.02–1.78)		0.22 (0.02–2.62)	
	Overdom	G/G-A/A	520 (93.4)	956 (95.8)	1.00	**0.04**	1.00	**0.02**
		A/G	37 (6.6)	42 (4.2)	**0.62 (0.39–0.97)**		**0.55 (0.33–0.92)**	
TLR8	Gen	G/G	314 (56.4)	492 (49.3)	1.00	**0.02**	1.00	0.24
		A/G	168 (30.2)	337 (33.8)	**1.28 (1.01–1.62)**		1.14 (0.88–1.49)	
		A/A	75 (13.5)	169 (16.9)	**1.44 (1.06–1.95)**		1.33 (0.93–1.90)	
	Dom	G/G	314 (56.4)	492 (49.3)	1.00	**<0.01**	1.00	0.14
		A/G-A/A	243 (43.6)	506 (50.7)	**1.33 (1.08–1.64)**		1.20 (0.94–1.52)	
	Rec	G/G-A/G	482 (86.5)	829 (83.1)	1.00	0.07	1.00	0.17
		A/A	75 (13.5)	169 (16.9)	1.31 (0.98–1.76)		1.27 (0.90–1.78)	
	Overdom	G/G-A/A	389 (69.8)	661 (66.2)	1.00	0.14	1.00	0.57
		A/G	168 (30.2)	337 (33.8)	1.18 (0.94–1.48)		1.08 (0.83–1.39)	

^*^Adjusted by gender, age, BMI, smoking, alcohol consumption, and diabetes; A-OR, adjusted odds ratio; CI, confidence interval; C-OR, crude odds ratio; Dom, dominant; Gen, genotypic; IFG, interferon gamma; IL1B, interleukin 1 beta; MARCO, macrophage receptor with collagenous structure; NOS2, nitric oxide synthase 2; Overdom, overdominant; Rec, recessive; TLR2, toll-like receptor 2; TLR8, toll-like receptor 8; VDR, vitamin D receptor. A statistically significant values of *p* and Odds ratios are indicated as bold. Boldness of these indicators are needed to highlight the important indicators.

TaqI and ApaI polymorphisms of the VDR gene, IL1B, and IFG did not show a statistically significant association with PTB in the overall group. FokI and BsmI polymorphisms of the VDR gene showed no significant association with PTB in the unadjusted model. However, after adjustment, their significance entered 0.05 (FokI, OR = 1.29, 95% CI = 1.02–1.63, *p* = 0.04; BsmI, OR = 1.14, 95% CI = 0.90–1.45, *p* = 0.02) in overdominant model, suggesting increased risk of PTB in individuals with heterozygous alleles (A/G for FokI, C/T for BsmI). Polymorphisms of TLR8 and NOS2 genes showed a statistically significant association with PTB in unadjusted analysis. However, in the adjusted model, *p* values of both polymorphisms exceed 0.05 (*p* = 0.24, and 0.08, respectively). MARCO gene polymorphism (rs2278589) showed a statistically significant association with an increased risk of PTB in several models in the overall study group.

Polymorphism of the TLR2 gene showed a decreased risk of PTB in the studied group. However, only three individuals in the case group and a single individual in the control group had a homozygous minor allele (A/A). The association analysis of this SNP with PTB requires larger cohort studies. Polymorphism of the MARCO gene showed a statistically significant association with PTB in several unadjusted models, and its significance remained after adjustment. A/G and G/G genotypes (OR = 1.57, 95% CI = 1.15–2.14, and OR = 1.73, 95% CI = 0.59–5.10, respectively, *p* = 0.01) increase the risk of PTB in the studied population. The overdominant model indicated the increased risk of PTB in individuals with heterozygous A/G allele (OR = 1.55, 95% CI = 1.14–2.12, *p* < 0.01).

Regarding to genotype distribution phenomenon, some genotypes are present in different proportions in different populations and lead to heterogeneity of genetic data. We analyzed an association of studied polymorphisms and risk of PTB separately in Asians and Caucasians, and males and females. Statistically significant associations are presented in Table 4.

**Table 4:** Association between PTB and studied polymorphisms in Asians and Caucasians.

Polymorphisms	Model	Genotype	Case, *n* (%)	Control, *n* (%)	C-OR (95 CI)	*p*-value	A-OR (95 CI)^*^	*p*-value
Asians (*n* = 1,242)								
VDR FokI	Overdom	G/G-A/A	261 (58.4)	427 (53.7)	1.00	0.11	1.00	**0.04**
		A/G	186 (41.6)	368 (46.3)	1.21 (0.96–1.53)		**1.32 (1.02–1.72)**	
VDR BsmI	Dom	C/C	263 (58.8)	434 (54.6)	1.00	0.15	1.00	**0.045**
		C/T–T/T	184 (41.2)	361 (45.4)	1.19 (0.94–1.50)		**1.31 (1.00–1.71)**	
MARCO	Gen	A/A	361 (80.8)	586 (73.7)	1.00	**0.02**	1.00	**0.01**
		A/G	80 (17.9)	196 (24.6)	**1.51 (1.13–2.02)**		**1.64 (1.17–2.30)**	
		G/G	6 (1.3)	13 (1.6)	**1.33 (0.50–3.54)**		**1.72 (0.58–5.10)**	
	Dom	A/A	361 (80.8)	586 (73.7)	1.00	**<0.01**	1.00	**<0.01**
		A/G-G/G	86 (19.2)	209 (26.3)	**1.50 (1.13–1.99)**		**1.65 (1.19–2.28)**	
	Overdom	A/A-G/G	367 (82.1)	599 (75.3)	1.00	**<0.01**	1.00	**<0.01**
		A/G	80 (17.9)	196 (24.6)	**1.50 (1.12–2.01)**		**1.62 (1.16–2.27)**	
NOS2	Gen	C/C	223 (49.9)	459 (57.7)	1.00	**0.01**	1.00	**0.04**
		C/T	183 (40.9)	287 (36.1)	**0.76 (0.60–0.97)**		**0.81 (0.62–1.07)**	
		T/T	41 (9.2)	49 (6.2)	**0.58 (0.37–0.91)**		**0.54 (0.33–0.89)**	
	Dom	C/C	223 (49.9)	459 (57.7)	1.00	**<0.01**	1.00	**0.04**
		C/T–T/T	224 (50.1)	336 (42.3)	**0.73 (0.58–0.92)**		**0.76 (0.59–0.99)**	
	Rec	C/C-C/T	406 (90.8)	746 (93.8)	1.00	0.053	1.00	**0.04**
		T/T	41 (9.2)	49 (6.2)	0.65 (0.42–1.00)		**0.59 (0.36–0.96)**	
TLR8	Gen	G/G	304 (68)	481 (60.5)	1.00	0.61	1.00	**0.04**
		A/G	127 (28.4)	265 (33.3)	1.32 (1.02–1.70)		**1.29 (0.96–1.72)**	
		A/A	16 (3.6)	49 (6.2)	1.94 (1.08–3.47)		**1.95 (1.03–3.70)**	
	Dom	G/G	304 (68)	481 (60.5)	1.00	0.60	1.00	**0.03**
		A/G-A/A	143 (32)	314 (39.5)	1.39 (1.09–1.77)		**1.37 (1.04–1.80)**	
Asian males (*n* = 597)^**^								
VDR FokI	Rec	G/G-A/G	215 (87)	313 (89.4)	1.00	0.37	1.00	**0.03**
		A/A	32 (13)	37 (10.6)	0.79 (0.48–1.31)		**0.53 (0.30–0.95)**	
VDR TaqI	Rec	A/A-A/G	232 (93.9)	342 (97.7)	1.00	**0.02**	1.00	**0.045**
		G/G	15 (6.1)	8 (2.3)	**0.36 (0.15–0.87)**		**0.38 (0.15–1.00)**	
TLR8	Gen	G/G	177 (71.7)	226 (64.6)	1.00	0.1	1.00	**0.044**
		A/G	61 (24.7)	100 (28.6)	1.28 (0.88–1.87)		**1.19 (0.77–1.84)**	
		A/A	9 (3.6)	24 (6.9)	2.09 (0.95–4.61)		**2.84 (1.18–6.82)**	
	Rec	G/G-A/G	238 (96.4)	326 (93.1)	1.00	0.1	1.00	**0.02**
		A/A	9 (3.6)	24 (6.9)	1.95 (0.89–4.26)		**2.71 (1.14–6.47)**	
Asian females (*n* = 645)^**^								
VDR BsmI	Gen	C/C	126 (63)	236 (53)	1.00	**0.033**	1.00	**0.017**
		C/T	67 (33.5)	179 (40.2)	**1.43 (1.00–2.03)**		**1.58 (1.04–2.40)**	
		T/T	7 (3.5)	30 (6.7)	**2.29 (0.98–5.36)**		**2.77 (1.07–7.15)**	
	Dom	C/C	126 (63)	236 (53)	1.00	**0.02**	1.00	**<0.01**
		C/T–T/T	74 (37)	209 (47)	**1.51 (1.07–2.12)**		**1.70 (1.14–2.54)**	
MARCO	Gen	A/A	160 (80)	318 (71.5)	1.00	0.056	1.00	**0.016**
		A/G	36 (18)	118 (26.5)	1.65 (1.08–2.51)		**2.03 (1.23–3.35)**	
		G/G	4 (2)	9 (2)	1.13 (0.34–3.73)		**1.59 (0.39–6.42)**	
	Dom	A/A	160 (80)	318 (71.5)	1.00	**0.02**	1.00	**<0.01**
		A/G-G/G	40 (20)	127 (28.5)	**1.60 (1.07–2.39)**		**1.99 (1.23–3.21)**	
	Overdom	A/A-G/G	164 (82)	327 (73.5)	1.00	**0.017**	1.00	**<0.01**
		A/G	36 (18)	118 (26.5)	**1.64 (1.08–2.50)**		**2.01 (1.22–3.31)**	
TLR8	Dom	G/G	127 (63.5)	255 (57.3)	1.00	0.14	1.00	**0.049**
		A/G-A/A	73 (36.5)	190 (42.7)	1.30 (0.92–1.83)		**1.50 (1.00–2.25)**	
Kazakhs (Asians, *n* = 1,186)								
MARCO	Gen	A/A	344 (80.4)	553 (73)	1.00	**0.02**	1.00	**<0.01**
		A/G	78 (18.2)	192 (25.3)	**1.53 (1.14–2.06)**		**1.60 (1.16–2.19)**	
		G/G	6 (1.4)	13 (1.7)	**1.35 (0.51–3.58)**		**1.08 (0.39–2.95)**	
	Dom	A/A	344 (80.4)	553 (73)	1.00	**<0.01**	1.00	**<0.01**
		A/G-G/G	84 (19.6)	205 (27)	**1.52 (1.14–2.02)**		**1.55 (1.14–2.11)**	
	Overdom	A/A-G/G	350 (81.8)	566 (74.7)	1.00	**<0.01**	1.00	**<0.01**
		A/G	78 (18.2)	192 (25.3)	**1.52 (1.13–2.04)**		**1.59 (1.16–2.19)**	
Caucasians (*n* = 313)								
VDR ApaI	Rec	C/C-A/C	92 (83.6)	147 (72.4)	1.00	**0.023**	1.00	**0.023**
		A/A	18 (16.4)	56 (27.6)	**1.95 (1.08–3.52)**		**1.98 (1.08–3.64)**	
Caucasian males (*n* = 129)^**^								
TLR8	Overdom	A/A-G/G	45 (73.8)	61 (89.7)	1.00	**0.017**	1.00	**0.017**
		A/G	16 (26.2)	7 (10.3)	**0.32 (0.12–0.85)**		**0.27 (0.09–0.84)**	

^*^Adjusted by gender, age, BMI, smoking, alcohol consumption, and diabetes; ^**^gender was excluded from adjustment model; A-OR, adjusted odds ratio; CI, confidence interval; C-OR, crude odds ratio; Dom, dominant; Gen, genotypic; IFG, interferon gamma; IL1B, interleukin 1 beta; MARCO, macrophage receptor with collagenous structure; NOS2, nitric oxide synthase 2; Overdom, overdominant; Rec, recessive; TLR2, toll-like receptor 2; TLR8, toll-like receptor 8; VDR, vitamin D receptor. A statistically significant values of *p* and Odds ratios are indicated as bold. Boldness of these indicators are needed to highlight the important indicators.

ApaI polymorphism of the VDR gene and polymorphism of the IL1B gene did not show a statistically significant association in Asians (males and females). FokI polymorphism of the VDR gene showed an increased risk of susceptibility to PTB in the overdominant model (G/G-A/A vs. A/G, OR = 1.32, 95% CI = 1.02–1.72, *p* = 0.04) in all Asian participants. However, this polymorphism showed a decreased risk of susceptibility to PTB among Asian males in the recessive model (A/A-A/G vs. G/G, OR = 0.38, 95% CI = 0.15–1.00, *p* = 0.045). TaqI polymorphism of the VDR gene showed a decreased risk of susceptibility to PTB only among Asian males in the recessive model (G/G-A/G vs. A/A, OR = 0.53, 95% CI = 0.30–0.95, *p* = 0.03). BsmI polymorphism of the VDR gene showed an increased risk of susceptibility to PTB among Asians in the dominant model (C/C vs. C/T–T/T, OR = 1.31, 95% CI = 1.00–1.71, *p* = 0.045). This polymorphism showed a strong association with PTB among Asian females in genotypic or codominant (C/C vs. C/T vs. T/T, OR = 1.58, 95% CI = 1.04–2.40 for C/T and OR = 2.77, 95% CI = 1.07–7.15, *p* = 0.017 for C/T, *p* = 0.017) and dominant (C/C vs. C/T–T/T, OR = 1.70, 95% CI = 1.14–2.54, *p* < 0.01) models. Interestingly, the minor allele (G) of rs2278589 was found only in 19 Asian individuals (1.5%) of our study population. rs2278589 polymorphism showed a statistically significant association with an increased risk of PTB in several models in overall Asians and Asian females but not in Asian males. Moreover, the deeper analysis of genotypes revealed that the minor G allele was detected only in ethnic Kazakhs (1.6%). Further analysis showed that MARCO gene polymorphism (rs2278589), and its heterozygous A/G genotype is associated with an increased risk of PTB in Kazakhs (A/A-G/G vs. A/G, OR = 1.59, 95% CI = 1.16–2.19, *p* < 0.01).

Polymorphism of the NOS2 gene showed a decreased risk of PTB among Asians and Asian males, but not in Asian females. A/A and A/G polymorphisms of the TLR8 gene showed a statistically significant association with PTB in overall Asians (OR = 1.95, 95% CI = 1.03–3.70, and OR = 1.29, 95% CI = 0.96–1.72, *p* = 0.04, respectively) and Asian males (OR = 2.84, 95% CI = 1.18–6.82, and OR = 1.19, 95% CI = 0.77–1.84, *p* = 0.044, respectively), but not in females. Interestingly, polymorphism of the TLR8 gene showed a statistically significant association with PTB in dominant model for overall Asians (G/G vs. A/G-A/A, OR = 1.37, 95% CI = 1.04–1.80, *p* = 0.03) and Asian females (G/G vs. A/G-A/A, OR = 1.50, 95% CI = 1.00–2.25, *p* = 0.049), but not for Asian males. The recessive model for TLR8 polymorphism revealed a statistically significant association with PTB among Asian males only, indicating that the A/A genotype increases susceptibility to PTB among Asian males (G/G-A/G vs. A/A, OR = 2.71, 95% CI = 1.14–6.47, *p* = 0.02).

A/A genotype of the TLR2 gene was detected only in one individual per group (case and controls) in Caucasians. Estimation of their association with PTB requires large cohort studies. FokI, TaqI, and BsmI polymorphisms of the VDR gene and polymorphisms of IFG and NOS2 genes did not show a statistically significant association among Caucasian representatives of the study group. An association analysis among Caucasian participants revealed a statistically significant association of ApaI polymorphism of the VDR gene with higher PTB risk in the recessive model, indicating that the A/A genotype almost doubles the susceptibility to PTB (C/C-A/C vs. A/A, OR = 1.98, 95% CI = 1.08–3.64, *p* = 0.023) in overall Caucasians, but not in Caucasian males and females separately. TLR8 genotypes showed a decreased risk of PTB in Caucasian males in the overdominant model (A/A-G/G vs. A/G, OR = 0.32, 95% CI = 0.09–0.84, *p* = 0.017). No significant association between the studied genotypes and risk of PTB was found in Caucasian females.

## Discussion

Tuberculosis remains a major health problem globally. The incidence rate has been decreasing over the years worldwide. However, incidence rates still hold high numbers in low- and middle-income countries where social and environmental factors dramatically contribute to the incidence number (1).

Kazakhstan is a multinational state with wide ethnocultural, linguistic, religious, racial, and national diversity, and is the ninth largest country by surface area (2.717 million km^2^) and takes 184th place in the world by population density. The population of Kazakhstan is over 20 million people and ethnic Kazakhs represent over 70% of the total population. Other nationalities include Russians (15.6%), Uzbeks (3.2%), Ukrainians (2.0%), Uighurs (1.5%), Germans (1.2%), Tatars (1.1%), and other ethnic groups and those who did not indicate nationality (5.1%) (39, 40).

Approximately 80% of the population of Kazakhstan consists of Asian ethnic groups. Caucasian ethnicities represent little less than 20%. The ratio of Asians and Caucasians in both case and control groups of our study population was the same (4:1). We had a response rate of 59.4% in the case group and 96.3% in the controls. The differences in response rates resulted in uneven representation of participants in case and control groups and could subjected to a selection bias. Response rate depends on many factors. Recent research works show that educated, employed, and economically stable persons are keener to participate in the studies or take a survey. Also, there is a lower chance that men will agree to participate in the case–control or epidemiologic study than women. 53.3% of recruited individuals were women which corresponds to recent response rate data (41). However, we recruited more men diagnosed with primary PTB than women. This confirms that the incidence rate among men is higher than among women, in Kazakhstan and globally (1). A low response rate in the case groups is normal since being diagnosed with a disease or health condition has a strong emotional effect, and we recruited patients right after hospitalization and before they started their treatment. In addition, there is a decline in response rates of case–control or epidemiologic studies, and people are reluctant to participate in the study (41). The response rate of 97.9% in the control group means healthy people are more likely to agree to participate in such studies.

Since social and environmental risk factors play a crucial role in TB incidence in low- and middle-income countries, Kazakhstan is not an exception. In the current research, we investigated an association of risk factors indicated by WHO with the PTB in Kazakhstan. We did not find any association between the PTB and such risk factors as smoking and regular alcohol consumption. There was no statistically significant association between PTB and residency (urban vs. rural). We could not estimate the role of contact with TB patients, HIV coinfection, drug abuse, pregnancy, and maternity leave due to the lack or low number of participants with these risk factors in the control group. However, underweight BMI (<18.49 kg/m^2^), diabetes, and unemployment showed a statistically significant association with PTB in our study group. Our findings confirm the results of other studies in different countries (42–44).

The disease progression does not depend on social and environmental factors alone. Genes regulate almost all processes in the human body. The immune response is a complicated process of interactions between host and pathogen where proper signaling and fast coordination take the central stage (15, 45). In the current study, we investigated the potential associations between functionally relevant polymorphisms of VDR (TaqI, ApaI, BsmI, and FokI), IL1B, IFG, MARCO, NOS2, TLR2, and TLR8 genes, and PTB in Kazakhstani population group. No significant associations were observed between IL1B and IFG polymorphisms and PTB risk. We could detect the A/A genotype of the TLR2 polymorphism (rs1898830) only in 4 individuals and the A allele constituted only 0.3% of our study population. However, according to the National Library of Medicine, the lowest frequency of the A allele (rs1898830) was detected in South Asians (over 40%), and the share of this allele in the global population is over 66% (46). These findings suggest a unique distribution of genotypes of TLR2 rs1898830 polymorphism among the Kazakhstani population. However, larger cohort studies are needed to identify the correct distribution of genotypes of this SNP and its association with PTB.

The association of rs2779248 polymorphism of the NOS2 gene with PTB is poorly studied. Velez et al. (47) showed an association of several NOS2 SNPs with TB. Gómez et al. (48) showed no individual association of the NOS2A gene with TB in the Colombian population. Möller et al. (49) reported that genes iNOS and CCL2 play a role in susceptibility to tuberculosis in the South African population. Our results showed an association of NOS2 gene polymorphism (rs2779248) with the lower risk of PTB in Asians.

TLR8 is an intracellularly expressed toll-like receptor and is located in the membranes of the endosomal compartment. It recognizes foreign single-stranded RNA and regulates the induction of IFN and inflammatory cytokines (50). The TLR8 gene locus encodes for two splice variants with alternative translation start sites, resulting in two variants of TLR8 protein that differ by 19 amino acids in the N-terminus, the functional contribution of which is unknown. The TLR8 rs3764880 SNP is located in the start codon region and regulates the translation of the two main TLR8 isoforms (51). In our study, rs3764880 polymorphism of the TLR8 gene did not show a significant association with PTB in the overall study group. The distribution of this polymorphism varies in many populations. The frequency of the A allele of the TLR8 polymorphism (rs3764880) is over 70% among Caucasians and less than 20% in Asians, so the heterogeneity of the genetic data was the reason for the lack of significance in the overall studied cohort. However, this polymorphism showed a statistically significant association with increased risk of PTB in the codominant model in overall Asians and Asian males but not Asian females. The combination of A/G and A/A genotypes was also associated with an increased risk of PTB in overall Asians and Asian females but not in Asian males. The A/A genotype of TLR8 polymorphism showed an association with an increased risk of PTB among Asian males in the recessive model (G/G-G/A vs. A/A) indicating that A/A polymorphism of the TLR8 gene increases the susceptibility to PTB almost three-fold in Asian males (OR = 2.71, 95% CI = 1.14–6.47, *p* = 0.02). In addition, the A/G heterozygous genotype showed an association with the decreased risk of PTB among Caucasian males but not in overall Caucasians and Caucasian females. An association between the TLR8 gene (rs3764880) and susceptibility to TB was shown in many populations. Zhou et al. (52) found an association of TLR8 polymorphisms with TB in the overall population. Wang et al. (50) reported an association of TLR8 polymorphisms with TB in Chinese Han population. Dalgic et al. (53) found an association of TLR8 polymorphism (rs3764880) with TB in Turkish male children. Varzari et al. (54) also reported an association of TLR8 polymorphisms with TB in Moldavian males. Davila et al. (55) reported a strong allelic association with the minor allele A of the rs3764880 with susceptibility to pulmonary TB in Indonesian and Russian males. All the abovementioned research works confirm the findings of our pilot (56) and current studies suggesting that the A/A genotype of TLR8 polymorphism (rs3764880) is strongly associated with TB risk in Asian males and can be a possible biomarker for PTB.

Macrophage receptor with collagenous structure (MARCO) is a member of the class A scavenger receptor family. MARCO is expressed on the cell surface of macrophages and binds invading intruders. It plays an important role in phagocytosis and activation of the immune response at early stages of infection (34, 57). MARCO gene has several SNPs and one of them, rs2278589 showed an association with TB in several populations. Ma et al. (58) reported on the association of two MARCO SNPs with TB in the Chinese Han population. Six years later Lao et al. (59) found an association of SNPs in MARCO and CD36 genes with TB in the Chinese Han population. Bowdish et al. (60) found an association of genetic variants of MARCO with PTB susceptibility in the Gambian population. Thuong et al. (34) found that rs2278589 was associated with susceptibility to TB and Beijing lineage of MTB in the Vietnamese population. The absence of the G allele in Caucasians and other Asians in our study group gives rise to several questions and requires larger cohort studies since this allele is present in over three-quarters of the global population (61).

Vitamin D is a strong immune modulator and is a key member of the immune response chain (15). The active form of vitamin D binds with its receptor, enters the nucleus, and activates the macrophages. This process also induces the synthesis of several antimicrobial agents. Many VDR gene polymorphisms have been tested for an association with TB. The most extensively researched ones are TaqI (rs731236), FokI (rs2228570), ApaI (rs7975232), and BsmI (rs1544410). Areeshi et al. (62) reported that variant allele A of FokI polymorphism showed an increased risk of PTB in Asians. Yadav et al. (63) also found a significant association of FokI with TB susceptibility in the Asian population. Chen et al. (24) reported an association of the variant homozygote genotype of the FokI polymorphism with a significantly increased risk of tuberculosis in the recessive model in the Chinese population [ff vs. Ff + FF: OR = 1.97, 95% CI: 1.32–2.93, *p* = 0.0032; heterogeneity test: χ(2) = 0.24, *p* = 0.62], but his finding showed a significantly decreased risk of tuberculosis for European subjects [bb + Bb vs. BB: OR = 0.41, 95% CI, 0.22–0.76, *p* = 0.02; heterogeneity test: χ(2) = 2.59, *p* = 0.11] in dominant model. However, our study revealed that the A/G genotype of FokI polymorphism of the VDR gene is associated with an increased risk of PTB in the overall group and Asians, but the A/A genotype was associated with a lower PTB risk among Asian males. Lee et al. (64) for a significant association of TaqI and BsmI polymorphisms with TB susceptibility in the Han Taiwanese population. Mohammadi et al. (65) investigated the association of FokI, TaqI, ApaI, and BsmI polymorphisms of the VDR gene with susceptibility to pulmonary tuberculosis in an Iranian population. He indicated that TaqI showed a significant association with the increased risk of TB in all models. BsmI showed a significant positive effect on TB risk only in its dominant genotype (bb + bB/BB) [1.44 (1.0, 1.9); *p*-value: 0.02]. FokI and ApaI did not show any significant effects on TB development in Iranian populations in their study (65). The recessive model analysis of the association between the ApaI polymorphism of the VDR gene and PTB among Caucasians showed that the A/A genotype increases susceptibility to PTB in Caucasians almost twice. Areeshi et al. (66) reported that ApaI polymorphism of the VDR gene is associated with a decreased risk of PTB in the overall, and African population.

To date, there is not much research done on the genetic component of susceptibility/predisposition to TB in the population of Kazakhstan. Zhabagin et al. (67) found a potential association between ApaI and FokI polymorphisms of the VDR gene and TB in Kazakh individuals of Almaty and Almaty area (south-east Kazakhstan). Sadykov et al. (25) performed an association study in vitamin D pathways with susceptibility to TB in Kazakhstan. They reported that FokI and BsmI polymorphisms of the VDR gene were associated with a decreased risk of TB (25). In our pilot study, we indicated that the heterozygous A/G genotype of the TLR8 gene was associated with an increased risk of PTB development in Kazakhs (56). Zhetkenev et al. (68) performed a preliminary case–control study in a Kazakhstani population. They found a statistically significant association between heterozygous rs12722 SNP polymorphism of the COL5A1 gene and TB susceptibility.

Our study presents important novel genetic findings. We found an association between FokI (rs2228570) and BsmI (rs1544410) polymorphisms of the VDR gene and an increased risk of PTB. Our study results showed that the A/A genotype of the TLR8 gene (rs3764880) is significantly associated with an increased risk of PTB in Asians and Asian males. For the first time, an association of MARCO gene polymorphism (rs2278589) and PTB risk in the Kazakhstani population was studied. It is worth mentioning that the G allele of the rs2278589 polymorphism of the MARCO gene was detected only in Kazakhs and it increases PTB susceptibility in the representatives of Asian ancestry and Asian females. A significant association between BsmI (rs1544410) polymorphism of the VDR gene and PTB risk among Asian females is also being reported for the first time. Another novel finding is the significant association between VDR ApaI polymorphism and PTB susceptibility in the Caucasian population of Kazakhstan. However, any findings must be interpreted with caution. More scientific evidence is required to state any SNP as a biomarker and the results of the present study should be validated in an independent cohort. This is the first research work that studied the epidemiology and genetics of PTB in Kazakhstan on a relatively large cohort.

Some limitations in our study that we have to bear in mind when interpreting our findings. The differences in the response rates between case and control groups led to the uneven representation of recruited individuals in our study cohort. The low number of selected SNPs is another limitation since we could miss polymorphisms that might play a key role in the immune response. We could not detect whether were the recruited individuals of the control group latently infected by TB since all participants in both groups had been vaccinated with BCG.

## Conclusion

This is the first study that evaluated the epidemiology and genetics of PTB in Kazakhstan on a relatively large cohort. Social and environmental risk factors play a crucial role in TB incidence in Kazakhstan. Underweight BMI (<18.49 kg/m^2^), diabetes, and unemployment showed a statistically significant association with PTB in our study group. FokI (rs2228570) and BsmI (rs1544410) polymorphisms of the VDR gene can be used as possible biomarkers of PTB in Asian males. rs2278589 polymorphism of the MARCO gene may act as a potential biomarker of PTB in ethnic Kazakh individuals. BsmI (rs1544410) polymorphism of the VDR gene and rs2278589 polymorphism of the MARCO gene can be used as possible biomarkers during the estimation of PTB risk in Asian females as well as ApaI polymorphism of the VDR gene in Caucasians.

## Data availability statement

The original contributions presented in the study are included in the article/Supplementary material, further inquiries can be directed to the corresponding authors.

## Ethics statement

The studies involving humans were approved by Local Ethics Committee of the Private Institution “National Laboratory Astana” (01–2020, June 26 2020). The studies were conducted in accordance with the local legislation and institutional requirements. The participants provided their written informed consent to participate in this study.

## Author contributions

DY: Conceptualization, Data curation, Formal analysis, Funding acquisition, Investigation, Methodology, Validation, Writing – original draft, Writing – review & editing. AG: Formal analysis, Investigation, Methodology, Writing – original draft, Validation. AAkh: Formal analysis, Investigation, Methodology, Validation, Writing – original draft. UK: Conceptualization, Investigation, Methodology, Writing – review & editing. SR: Conceptualization, Data curation, Investigation, Validation, Writing – review & editing. UK: Data curation, Investigation, Software, Writing – review & editing. GZ: Formal analysis, Investigation, Methodology, Writing – original draft. RK: Data curation, Software, Writing – review & editing. AAki: Conceptualization, Data curation, Investigation, Methodology, Writing – review & editing.
