# Supplementary material for: Pulmonary tuberculosis epidemiology and genetics in Kazakhstan
Source: Front Public Health. 2024 Apr 19;12:1340673. doi: 10.3389/fpubh.2024.1340673 (PMC11066200; doi:10.3389/fpubh.2024.1340673)
Supplement: Supplementary file 1 [file Data_Sheet_1.PDF]

MOLECULAR-GENETIC RESEARCH QUESTIONNAIRE

ID number ____________________________
Full name ____Sex _________________
Examination date "__"_______________20___ Nationality _______________________________
Birth date "__"________________________ Age _________________
Weight at the time of examination_________kg Height ________cm
Significant weight changes (+ ________kg; - ___kg) noted over the last weeks, months, years (underline as appropriate). Profession____
Normal blood pressure (BP) in numbers____
Figures of maximum BP and from what age an increase has been noted________________________
If there is an increase in BP, what medications are used_____________________________________
Increase in cholesterol, triglycerides, high (low) density lipoproteins (underline as appropriate), from what age (if possible, indicate the level of)? _______________________________________
____
Elevated bilirubin level: yes, no, from what age (if possible, indicate the level of total, direct and indirect bilirubin)____
____
Has there been an increase in homocysteine levels: yes, no, since what age (if possible, indicate the level)?)
____
Have you ever followed a diet and what kind of diet (restriction of what foods, duration, on your own or doctor's orders)? ____
____
Smoking (since what year, how many cigarettes per day?) ___________________________________
Alcohol consumption: yes, no, how often?____
Do chest pains bother you: yes, no; how are the attacks alleviated? ___________________________ ,
frequency of episodes________________________; are the pains associated with emotional or physical stress; other____
Myocardial infarction in the medical history: yes, no, if yes, at what age?_____________________
Stroke in the medical history____
Pain along veins in the legs
Varicose veins of the lower limbs, esophagus, rectum (underline as appropriate): yes, no; from what age?
____
Increased blood clotting (thrombophilia/hypercoagulation): yes, no; from what age?
____
History of thrombosis: yes, no; from what age?____
Current/planed therapy for thrombophilia/thrombosis (specify medications and dosages)____________________
____
Myocardial infarction, stroke, ischemic heart disease, arterial hypertension, atherosclerosis, thrombosis (underline as appropriate) in first and second degree relatives? If yes, indicate which of them
____
Type I or II diabetes mellitus (underline as appropriate) from what age? ______________, fasting glucose level___________, medicines used ____
Type I or II diabetes mellitus (underline as appropriate) in first and second degree relatives? If yes, specify who ____
Osteoporosis: From what age?________, medications used ________________________________
Osteoporosis in first and second-degree relatives? If yes, specify who among them____________
Allergic diseases: yes, no; how do they manifest?_____________________________________
Do first- and second-degree relatives have allergic diseases? If yes, specify which ones and whom
____

Suspected presence of Alzheimer`s disease: yes, no
Alzheimer`s disease in first and second-degree relatives? If yes, specify who _________________

Suspected presence of Crohn`s disease: yes, no
Crohn`s disease in first and second-degree relatives? If yes, specify in whom__________________
Oncological diseases: yes, no; diagnosis/localization/age___________________________________

____
Surgically operated, date, scope of the operation____
Treatment received (which medications, frequency of radiation and/or chemotherapy)

____
Oncological pathology in first- and second-degree relatives?
If yes, specify the type, in whom, and at what age it was detected____________________________

____

For women:

Menstruation since what age___________________, duration ____________________________,
regularity: yes, no; cycle duration_______________, painfulness: yes, no
Menopause yes, no, from what age____
Taking oral (hormonal) contraceptives yes, no, specify which ones__________________________
Gynecological diseases (specify which ones and since what age)_______________________________

____
Change in hormonal status: yes, no; specify which hormones are increased/decreased

____
Spontaneous miscarriages/unintended pregnancies: yes, no
If so, how much________, at what gestational age_______________________________________
Complications during previous pregnancies (gestosis/fetoplacental insufficiency) yes, no
What were the manifestations?____
Have you recently given birth or taken maternity leave?

- Yes, within the past 6 months
- Yes, within the past year
- Yes, more than a year ago
- No
- Prefer not to answer

For men:

Disruption of spermatogenesis: yes, no; how does it manifest?______________________________

For both sexes:

Parental status: yes, no
Infertility______years _____number of partners _________________________
Cases of children born with mental retardation, chromosomal anomaly or congenital malformations (underline as appropriate) in the genealogic table
If yes, which of the relatives is sick (is necessary to specify pathology)__________________________________
____
Consanguinity marriage: yes, no; what is the relationship between the spouses? ________________
Cases of cystic fibrosis, phenylketonuria, spinal muscular atrophy, nonsyndromal sensorineural hearing loss, adrenogenital syndrome, Gilbert`s syndrome (underline as appropriate) in the genealogic table.
If yes, which relative has the disease?____
Have you undergone any genetic testing before: yes, no
If yes, which one____
Which genetic test has been prescribed for the patient?_____________________________________

TB (tuberculosis) screening:

Have you ever been diagnosed with TB? yes, no. If yes, specify diagnosis/age ____________________
TB disease in first and second-degree relatives? If yes, specify in whom _________________________
Have you ever been vaccinated with BCG? : yes, no ____
Have you ever had close contact with persons known or suspected to have active TB disease? : yes, no
____
Do you have any chronic medical conditions (HIV/AIDS)? yes, no
Have you ever been a volunteer or healthcare worker who served clients who are at increased risk for active TB? yes, no If yes, where/when ____
Do you have a persistent cough lasting more than 3 weeks? yes, no __________________________________
Have you experienced unexplained weight loss? yes, no ____
Do you work or have you worked in a healthcare setting? yes, no ___________________________________
Do you have a stable income? Yes, no ____
Do you have access to the following goods in your home? (Check all that apply)

- ☐ Washing machine
- ☐ Refrigerator
- ☐ Microwave
- ☐ Television
- ☐ Computer or laptop
- ☐ Internet access
- ☐ Mobile phone
- ☐ Dishwasher
- ☐ Air conditioning
- ☐ Central heating
- ☐ Other (please specify) ____

Do you have access to running water in your home?

- ☐ Yes, hot and cold water
- ☐ Yes, cold water only
- ☐ No, no running water

Do you have access to enough food for an active and healthy lifestyle? yes, no
How would you describe your typical diet?

- ☐ Mostly home-cooked meals
- ☐ Mostly fast-food
- ☐ Vegetarian or Vegan

How often do you consume fruits and vegetables?

- ☐ Daily
- ☐ Several times a week
- ☐ Occasionally
- ☐ Rarely
- ☐ Never

How often do you consume meat?

- ☐ Daily
- ☐ Several times a week
- ☐ Occasionally
- ☐ Rarely
- ☐ Never

How often do you consume fish meat or sea food?

- ☐ Daily
- ☐ Several times a week
- ☐ Occasionally
- ☐ Rarely
- ☐ Never

What type of area do you live in?

- ☐ Urban
- ☐ Rural

Do you currently use illicit drugs or misuse prescription medications? yes, no
If yes, please specify the type(s) of drugs or medications used and frequency of use.

INFORMED CONSENT FOR MOLECULAR GENETIC STUDY

Dear research participant!

Following your desire (to participate), you are expected to take a sample of biological material (blood, buccal cells, biopsy material, etc.), which will be used for molecular genetic research (DNA research). These studies will help identify the carriage of certain genetic diseases or an increase in the risk of the most common diseases, the development of which depends on both hereditary and environmental factors.

Before a molecular genetic study is performed, the staff of the ______(ORGANIZATION NAME)________________, must explain to you the purpose of testing, its information content, the likelihood of obtaining the correct result, and the possible consequences of testing (including the possibility of practical use of the results) for you or your relatives.

By signing this form, you confirm that you are giving your consent to the molecular genetic testing voluntarily, with sufficient information, and that you have had the opportunity to ask any questions you may have regarding the molecular genetic testing. All personal data concerning you or your relatives is confidential and cannot be transferred to other persons except with your express consent. You can withdraw your consent at any time without further explanation.

I hereby express my consent that genetic testing will be performed:

Me ____
(Full Name) (Date of Birth)

with the aim
____

and biological material will be retained until results are obtained and evaluated, and for other additional studies to be performed if required to obtain clear results.

______________________________ ______________________________
(date) (Sign of a participant)

______________________________________ __________________________
(Name of laboratory employee) (Sign)

Please specify exactly how your samples may be used.

After the laboratory employee answers all your questions, answer the proposed questions by marking “YES” or “NO” in the appropriate boxes:

I agree that the samples will be used for the following molecular genetic study(s): ____________________	Yes	No
I want to know the results of molecular genetic research	Yes	No
I agree that the research results will be provided to:<br>• only me<br>• contact person<br>____________________(indicate)<br>• family member<br>____________________(indicate)	Yes	No
I agree that the sample may be used for scientific research, if this does not result in disclosure of personal information about me.	Yes	No
If necessary, research can be used in medical genetic counseling or genetic research of other family members.	Yes	No
I agree that, if necessary, the laboratory staff member conducting the study can contact me using the contact information.	Yes	No

I can change my mind on one or all points at any time.

____________________ ____________________ ____________________
(Full name of participant) (Date) (Sign)

Address ____

e-mail____________________

phone____________________

____________________ ____________________ ____________________
(Full name of laboratory employee) (Date) (Sign)

Genealogy – Genealogical Tree

If possible, indicate if anyone has/had any illnesses (age, outcome)

An example of drawing up a genealogical tree is presented below (the arrow indicates the proband, the person in relation to whom the proband, the person in relation to whom the genealogy is drawn up (the child in whom the study is conducted)), if there are diseases in relatives, write next to the diagnosis, the age of onset of the disease, outcome)

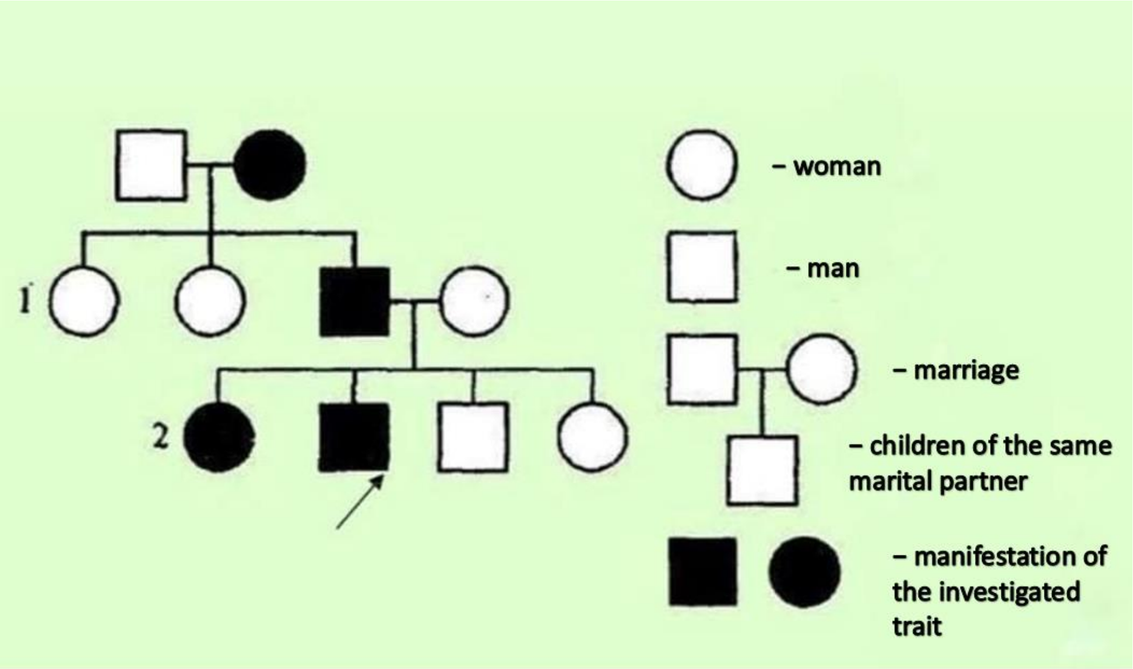

Additional information

Conclusions of Clinicians'

Examinations – ECG, EchoCG

And other copies if possible.

Biomaterial collection card for

Molecular-Genetic Study

Date of sampling ________________

ID ____

Sex _________________

Nationality ______________________________

Date of Birth "_____"_________________г.

Age _______________years

Weight at the time of examination _________kg

Height __________cm

BMI_____ kg/m^2

AP _______________________mmHg Chest.__cm, Waist _________________, cm,

Hips ___________cm, Blood type ________________

Biomaterial

N	Biomaterial	
1	Blood with K_2EDTA	
2	Blood with K_2EDTA	
3	Blood with clot activator	
4	Blood with clot activator	
5		
