# Supplementary material for: Pulmonary tuberculosis epidemiology and genetics in Kazakhstan
Source: Front Public Health. 2024 Apr 19;12:1340673. doi: 10.3389/fpubh.2024.1340673 (PMC11066200; doi:10.3389/fpubh.2024.1340673)
Supplement: Supplementary file 2 [file Data_Sheet_2.PDF]

Detailed information of predesigned TaqMan™ probes for Real-time Polymerase Chain Reaction

Gene	Name	Reference number	Context Sequence	Assay ID
VDR	FokI	rs2228570	GGAAGTGCTGGCCGCCATTGCCTCC **[A/G]** TCCCTGTAAGAACAGCAAGCAGGCC	C__12060045_20
	TaqI	rs731236	TGGACAGGCGGTCCTGGATGGCCTC **[A/G]** ATCAGCGCGGCGTCCTGCACCCCAG	C___2404008_10
	BsmI	rs1544410	GAGCAGAGCCTGAGTATTGGGAATG **[T/C]** GCAGGCCTGTCTGTGGCCCCAGGAA	C___8716062_20
	ApaI	rs7975232	AAGGCACAGGAGCTCTCAGCTGGGC **[A/C]** CCTCACTGCTCAATCCCACCACCCC	C__28977635_10
IL1B	A > G	rs16944	TACCTTGGGTGCTGTTCTCTGCCTC **[G/A]** GGAGCTCTCTGTCAATTGCAGGAGC	C___1839943_10
IFG	+874 A > T	rs2430561	Custom probe	Custom probe
MARCO	A > G	rs2278589	GGGGTGCAGTCCGCTAGTTGCAGCT **[A/G]** AGCATCTGGCTCTTTCCAAGGACTC	C__15965855_10
NOS2	C > T	rs2779248	TTCATCAGCAGGGTGGCTGCTAAGA **[C/T]** AGAGGCACCACGGAGCCAGGTTTTA	C___2593688_10
TLR2	G > A	rs1898830	ATAGTAAAATAAATCCAGAGAAATC **[A/G]** GAACAGGGGAAATAATAATATAAGA	C__11853988_10
TLR8	G > A	rs3764880	AATGAAAAATTAGAACAACAGAAAC **[A/G]** TGGTAAGCCACTTCTATTTCTTTAG	C___2183830_10

IFG; interferon gamma; IL1B: interleukin 1 beta; MARCO: macrophage receptor with collagenous structure; NOS2: nitric oxide synthase 2; TLR2: toll-like receptor 2; TLR8: toll-like receptor 8; VDR: vitamin D receptor
